# The visual amplification of goal-oriented movements counteracts acquired non-use in hemiparetic stroke patients

**DOI:** 10.1186/s12984-015-0039-z

**Published:** 2015-06-09

**Authors:** Belén Rubio Ballester, Jens Nirme, Esther Duarte, Ampar Cuxart, Susana Rodriguez, Paul Verschure, Armin Duff

**Affiliations:** Laboratory of Synthetic Perceptive, Emotive and Cognitive Systems, Center of Autonomous Systems and Neurorobotics, Pompeu Fabra, Roc Boronat, Barcelona, Spain; Servei de Medicina Física I Rehabilitació, Hospitals del Mar I l’Esperanç, Institut Hospital del Mar d’Investigacions Médiques, Barcelona, Spain; Servei de Medicina Física i Rehabilitació, Hospital Universitari Vall dHebron, Barcelona, Spain; ICREA, Institució Catalana de Recerca i Estudis Avançats, Passeig Lluís Companys, Barcelona, Spain

**Keywords:** Stroke rehabilitation, Hemiparesis, Upper extremity, Physical therapy, Learned non-use, Reinforcement-based motor therapy

## Abstract

**Background:**

Stroke-induced impairments result from both primary and secondary causes, i.e. damage to the brain and the acquired non-use of the impaired limbs. Indeed, stroke patients often under-utilize their paretic limb despite sufficient residual motor function. We hypothesize that acquired non-use can be overcome by reinforcement-based training strategies.

**Methods:**

Hemiparetic stroke patients (n = 20, 11 males, 9 right-sided hemiparesis) were asked to reach targets appearing in either the real world or in a virtual environment. Sessions were divided into 3 phases: baseline, intervention and washout. During the intervention the movement of the virtual representation of the patients’ paretic limb was amplified towards the target.

**Results:**

We found that the probability of using the paretic limb during washout was significantly higher in comparison to baseline. Patients showed generalization of these results by displaying a more substantial workspace in real world task. These gains correlated with changes in effector selection patterns.

**Conclusions:**

The amplification of the movement of the paretic limb in a virtual environment promotes the use of the paretic limb in stroke patients. Our findings indicate that reinforcement-based therapies may be an effective approach for counteracting learned non-use and may modulate motor performance in the real world.

**Electronic supplementary material:**

The online version of this article (doi:10.1186/s12984-015-0039-z) contains supplementary material, which is available to authorized users.

## Introduction

Following stroke, a loss of neural tissue induces drastic neurophysiological changes that often result in cognitive and motor impairments, such as hemiparesis. In order to counteract these deficits patients often introduce compensatory movements (e.g. overutilizing their non-paretic limb). Although these compensatory strategies may immediately improve functional motor performance in activities of daily living (ADLs) or reduce the burden of using the paretic limb, a long period of non-use of the affected limb can lead to further reversible loss of neural and behavioral function [1]. This so-called learned non-use has been associated with a reduced quality of life. Hence, methods must be found to reduce the impact of acquired non-use.

A possible treatment for learned non-use is Constraint Induced Movement Therapy (CIMT), which forces the patient to use the paretic limb by constraining the movement of the non-paretic limb. This technique has been shown to be effective in mitigating the effects of learned non-use [2–4]. However, due to the high intensity and long duration of CIMT protocols, which can range from 1 to 6 hours of training per session [5], they may reduce quality of life, affect the patient’s adherence to therapy, be prohibitively expensive and even inconvenient for those patients with severe motor or cognitive deficits [6]. Moreover, it remains unclear whether the standard CIMT protocols are more beneficial than bimanual functional rehabilitation [7]. The success rate of the standard CIMT protocols may depend on the severity of upper limb paresis and latency of intervention post-stroke. Consequently, its application remains restricted to subacute patients, with no severe cognitive impairments, and mild hemiparesis. These very stringent inclusion criteria only account for about 15 % of stroke cases [8]. Hence, in light of these limitations it seems opportune to develop rehabilitation techniques that build on the positive aspects of CIMT, i.e. enhanced use of the paretic limb, while mitigating the negative ones.

CIMT builds on the emergence of compensatory movements post-stroke. Such movements can be acquired and retained through the involvement of the mirror neuron system (MNS) [9]. For instance, it has been proposed that a successful action outcome might reinforce not only the intended action but also any movement that drives the MNS during the course of its execution [10–12]. Action selection may depend on both the action’s executability and desirability [13]. In this case, the executability of an action is modulated by the MNS and indicates the action’s expected biomechanical error, while the desirability of an action is modulated by its outcome and can be reinforced by accidental success. Desirability of action was demonstrated in a recent study exploring handedness bias, suggesting that performance asymmetries between limbs may influence the choices that individuals make about which hand to use [14]. A recent study on hemiparetic stroke patients proposed that increasing the patient’s confidence in using the paretic arm for a given level of function may be critical for recovering non-pathological hand selection patterns [15]. In this vein, Stoloff and colleagues showed that modulating reward rates during a reaching task can increase the use of the non-dominant hand in healthy subjects [16]. In order to ensure a high performance level the authors used a variable ratio staircase procedure to adjust the size of a virtual target when users selected their non-dominant limb to perform the action. The visual representation of the target remained fixed. Note however, that this reinforcement strategy exposes the user to an incomplete visuomotor feedback of the reaching action when performed using the dominant limb. This is because, due to the changing size of the virtual target, the movements executed with the non-dominant limb were inferior in extent and accuracy when compared to the dominant limb. We hypothesize that exposing a user to the complete visual feedback of an intended action may lead to reinforcement and thus will modulate hand selection patterns.

The approach we take towards stroke rehabilitation is based on the Distributed Adaptive Control (DAC) theory of mind and brain, which proposes that the disruption of the sensorimotor contingencies leads to a reduction of activity in the motor cortical pathways and interconnected sensorimotor areas. This reduction of activity leads to a reduced drive in neural plasticity and thus to an impairment of recovery in case of a lesion to the brain. Consequently, restoring these sensorimotor contingencies through external manipulation of sensory and/or motor modalities during the execution of goal-oriented actions might increase the activation of the MNS and its interconnected motor areas, thus driving plasticity and recovery [17]. On this basis, Virtual Reality (VR)-based protocols have proved beneficial in the clinical context, since they can provide multimodal feedback in safe and ecologically valid training environments. Indeed, we have demonstrated this using the so called Rehabilitation Gaming System (RGS) [18, 19] and provided direct evidence for the involvement of the MNS in these VR-based manipulations [20].

The aim of this study is to explore to what extent goal-oriented movement amplification in VR can induce beneficial changes in a patient’s hand selection patterns, namely, effectively reinforcing the use of the paretic limb, reversing learned non-use, and promoting motor recovery. To realize this, we instructed stroke patients to perform a reaching task in the first person VR RGS environment and a pointing task in the real world. We included in the RGS protocol movement amplification of the paretic limb. Results show that visual amplification of the movement of the virtual counterpart of the paretic limb may induce improvements in use of the affected arm and modulate performance in the real world.

## Methods

### Participants

Patients fulfilling the inclusion/exclusion criteria (see below) were first approached by an occupational therapist at the rehabilitation units of Hospital Esperança or Hospital Vall d’Hebron from Barcelona to determine their interest in participating in a research project on VR-based motor rehabilitation using the Rehabilitation Gaming System. Inclusion criteria were as follows: a) Upper limb hemiparesis secondary to a first-ever ischaemic stroke (MCA territory) or hemorrhagic stroke (intracerebral); b) Proximal upper limb motor deficit (Medical Research Council Scale for proximal muscle strength (MRC) >3); and c) Capable and willing to participate in the RGS therapy (Mini-mental state examination (MMSE) >22). The ethics committee of clinical research of the Parc de Salut Mar and Vall d’Hebron Research Institute approved experimental guidelines. In total 26 hemiparetic stroke patients were identified as potentially eligible participants. We excluded a further six participants showing a previous history of upper-limb motor disability and major cognitive deficits and seizures. The remaining 20 participants (n = 20, age = 62.2 ± 14.3, 11 males, 9 right-sided hemiparesis, MRC = 3.7 ± 0.5, MMSE = 26.8 ± 2.8, 17 ischaemic, 211 ± 390.9 days post stroke) were informed about the aim and procedures of the study, signed informed consent forms and were blinded to the experimental hypotheses.

### Design

**Fig. 1:**
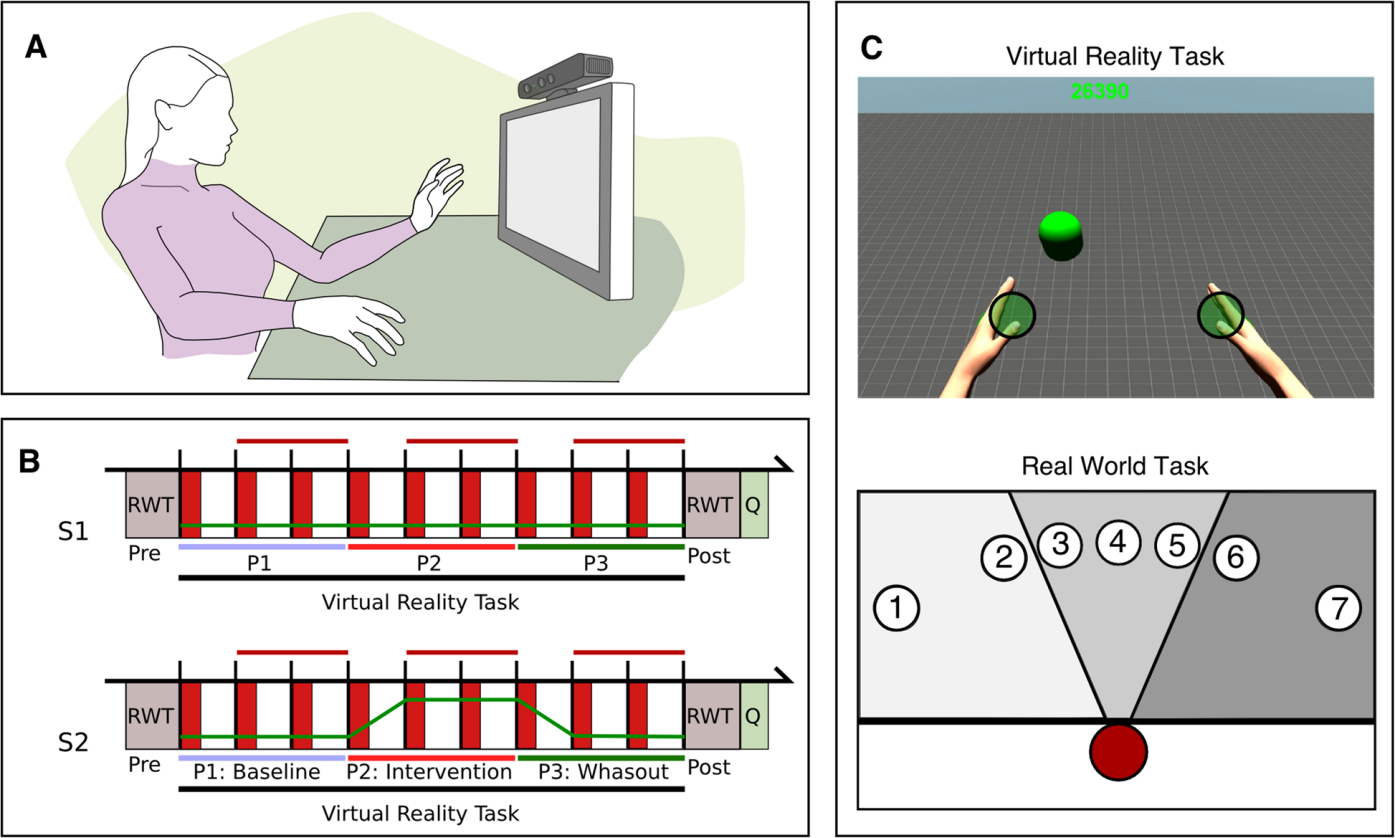
The RGS setup. **a**: Microsoft Kinect sensor captures the movements of the user’s upper limbs and maps them into an avatar displayed on a screen in first person perspective so that the user sees the upper extremities. **b**: The experimental protocol is divided in two sessions (S1 and S2) comprising a Real World Task (RWT), a Virtual Reality Task, and a Questionnaire (Q). The amplification of the virtual movement of the paretic limb (green line) is manipulated during the Virtual Reality Task, which is divided in 3 phases (P1, P2, and P3). Horizontal red lines indicate blocks of trials for which performance measurements are considered for analysis. Vertical red rectangles indicate forced trials. White rectangles indicate free choice trials. **c**: VR and RW tasks, top: participants performed consecutive reaching movements in VR, bottom: participants performed consecutive pointing movements towards targets located at different angles corresponding to the paretic, center, or non-paretic workspace

**Fig. 2:**
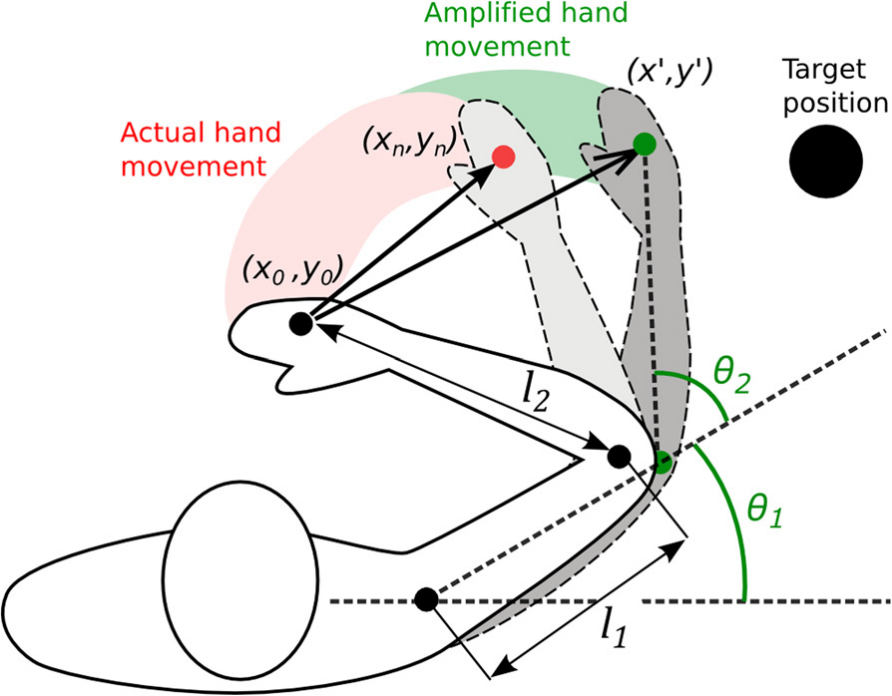
Methodology for the amplification of goal-oriented reaching movements in VR. The trajectory of the movement executed (red shadow) is amplified both in extent and accuracy (green shadow) towards the target position, deriving from the start position (*x*_0_,*y*_0_) and current position (*x*_*n*_,*y*_*n*_) of the actual movement

In order to study the potential of goal-oriented visuomotor amplification for promoting the use of the paretic limb, we use the Rehabilitation Gaming System (RGS) (Fig. 1a), which allows the user to control a virtual body (avatar) seen from a first-person perspective on a computer screen via their own movements that are captured by an imager at 30 Hz (Kinect, Microsoft). Physical execution of goal-directed movement is thus coordinated with the observation of the same movement in VR. RGS includes the Adaptive Biomechanics Controller, which modulates the task difficulty though the amplification of the movement of the virtual limb. Modulation of the movement is achieved by combining two methods: amplifying the amount of movement (i.e. extent amplification) and by attracting the direction of the movement towards the target position (i.e. accuracy amplification) (Fig. 2). Thus range of movement amplification reduces visual errors in movement extent, while accuracy amplification lessens visual directional errors relative to the target. In order to compute the position of the amplified virtual hand at each timeframe, we first extend the vector of the actual hand movement executed by the patient:

(1) $$ \textbf{m}_{e}= \mathbf{m} \cdot G  $$

where *m*_*e*_ is the vector of the extended hand movement, *m* is the actual hand movement with respect to the start position, and *G* is a constant ratio of extent amplification.

Next we project the amplified movement vector *m*_*e*_ onto the target direction:

(2) $$ \textbf{m}_{p}= (\hat{\textbf{t}} \cdot\textbf{m}_{e}) \hat{\mathbf{t}}  $$

where the operator · denotes a dot product, **t** is the distance vector from the start position to the target, and $\hat {\textbf {t}}$ is the unit vector of **t**.

Finally, the movement amplification at the current frame is defined by

(3) $$ {\textbf{m}_{a}}= \alpha\cdot{\textbf{m}_{p}} + (1-\alpha) {\textbf{m}_{e}}  $$

(4) $$ \textbf{where}~~ \alpha= \frac{|{\textbf{m}_{p}}|} {|\mathbf{t}|} \cdot \frac{1}{G}.  $$

The movement amplification vector **m**_*a*_ is a weighted combination of two terms: an accuracy amplification vector and an extent amplification vector. The *α* ratio determines the contribution of each of these two components, and will cancel the amount of amplification of the movement extent when the patient exceeds in distance the desired movement **t**. Contrarily, if the direction of the executed movement matches the target direction, *α* will approach 0, thus decreasing the amount of accuracy amplification. After computing the movement amplification vector **m**_*a*_ and extracting its corresponding hand position (*x*^′^,*y*^′^), we recursively applied an inverse kinematics technique (Cyclic Coordinate Descent) [21] for estimating the angles of elbow and shoulder joints of the avatar. The constant coefficient *G* was 1.4. The length of the segments of the avatar’s upper-limbs were *l*_1_=0.27, and *l*_2_=0.38. Notice that *l*_2_ denotes the distance from elbow to fingers and therefore exceeds the length of the forearm (Fig. 2).

We developed a new scenario in RGS to quantify and modulate effector selection in stroke patients. Participants were instructed to reach for targets that appeared consecutively in a virtual environment (Fig. 1c, top). At the beginning of each trial, subjects had to position both virtual hands over their corresponding start positions. Start positions were indicated by two green cylinders (7.5 cm diameter) centered 48 cm apart. After the subject maintained the avatar’s hands over the start positions during a variable time interval of 1 ±0.5 ms the two green cylinders disappeared and a target sphere appeared at any of nine possible angles (0°, ±4°, ±8°, ±16°, ±32°) along a semicircular array 65 cm from the projected center of the avatar. Trial time limits (1.75 s) were indicated by continuous changes in the color of the target, which ranged from green to black. Trial time limits were fixed according to the results from a pilot study with stroke patients to guarantee that patients were able to perform a complete reaching movement within this time window. At the end of the trial the target disappeared. The participants were instructed to reach the target as fast as possible with one hand and keep the other hand over the start position. Trials in which the participant moved both hands were automatically invalidated and immediately repeated. Movements in the virtual world were confined to the horizontal plane. Trunk movements in the virtual environment were constrained to ±30° axial rotation. When the center of the virtual hand was placed over the target, participants heard a continuous tone and could observe the increase of their score by 30 points every tenth of a second. These score values were permanently displayed at the top of the screen and accumulated across blocks and phases.

The study was divided into two sessions (Fig. 1b): a familiarization period (S1), and an experimental period (S2). Both sessions were completed during two consecutive days. A session comprised 2 blocks of 14 pointing RW trials each (pre and post phases in Fig. 1b), and 9 blocks of 32 VR-based reaching trials each. VR-based trials were divided in three phases (P1, P2, and P3). In session 2, we refer to these three phases as baseline, intervention and washout. Each of these three phases was divided into 3 blocks of 32 trials each. During the intervention phase we amplified the mapping of the physical movement of the paretic arm to the matched virtual limb. This amplification was progressively and uniformly introduced during the first block of the intervention phase and gradually reduced during the first block of the washout phase. We introduced and suppressed the visuomotor amplification in a gradual fashion to keep participants explicitly unaware of the manipulations.

After each block of trials the patient rested for twenty seconds. In the beginning of each block we included eight forced lateralized trials i.e. four trials with the non-paretic and with the paretic limb respectively, to ensure that participants experience the effect of the kinematic and goal-oriented amplification of the paretic limb. These forced trials where indicated to the subjects by the presentation of only one virtual limb and its corresponding initial position aligned with the position of the corresponding limb of the subject. In the following 24 free-choice trials patients could freely choose their preferred limb. Free choice trials were indicated by the presentation of both virtual limbs at their corresponding initial positions. Within each block of 24 free choice trials, targets appeared at the 0° location on four trials, at the ±4° location on eight trials, at the ±8° location on six trials, at the ±16° location on four trials, and at the ±32° location on two trials. This distribution gave us a higher resolution in the measurement of hand positions closer to the center of the task space therefore reducing ambiguity in assessing hand selection patterns. The sequence of target locations was randomized. The most lateral, ±32° locations, were only reachable by the ipsilateral limb, and were included to decrease the likelihood that participants would use only one hand to reach all the targets. These outer locations were excluded during forced trials.

In order to assess how the effects of visuomotor amplification in VR transfer to real world motor performance, participants performed a pointing task in the real world (Fig. 1c, bottom) before (pre-evaluation) and after (post-evaluation) completing the VR-based reaching task. Each of these evaluations consisted in 14 consecutive trials, the first 7 to be performed using the non-paretic limb and the last 7 using the paretic limb. The trials in which the patient used the non-paretic limb were introduced to guarantee that the task was well understood. In the beginning of each trial participants were instructed to place their index finger at the start position, centered 20 cm in front of them. Next, a colored number from 1 to 7 appeared on the screen and the participant had to point with the whole arm towards the corresponding target until achieving maximum extension. Targets were presented in a semicircular array of cards over the table, displaying colored numbers ordered from 1 to 7 at -32, -16, -8, 0, 8, 16 and 32 degrees, 1 m from the start position. Patients were instructed to self-pace their movements. After completing each pointing movement they returned to the start position. The order of presentation of each of the 7 pointing trials was randomized. Notice that in the real wold task we did not measure hand selection patterns and patients performed one pointing movement per angle and limb within each evaluation phase (pre and post). We took this decision regarding the experimental design in order to shorten its total duration and prevent fatigue.

### Outcome measures

In order to evaluate changes in hand selection patterns, we calculate the Point of Subjective Equality (PSE) [16] for each patient over the different phases. The PSE is the theoretical position in space where a subject shows equal probability of spontaneously choosing either limb to reach it. In order to estimate the PSE for each patient and phase we constructed a psychometric function of the probabilities of using the non-paretic/paretic limb to reach towards each target position. Next we fitted the resultant data points using logistic regression as described in [16]. Before estimating the PSEs, we normalized workspaces across subjects by mirroring target directions for those patients with their right arm affected.

To evaluate the participants’ perception of the movement manipulation, a 5-point Likert scale self-report questionnaire was administered at the end of each session (see Additional file 1). The questionnaire consisted of 18 items divided in three different categories referring to the controllability of the virtual arms, subjective competence, and effort, in relation to the paretic and the non-paretic limb. We used reverse polarity on 1/3 of the questions within each category in order to avoid response bias.

### Data analysis

To assess the overall within-subject impact of the treatment on effector selection patterns, we performed a 2-tailed Wilcoxon signed-rank test of the PSE values from the baseline, intervention, and washout phase. In order to study the effects of the patients’ reinforcement history on hand selection, we performed a sequential analysis of hand bias by running a two-way repeated-measures ANOVA on the probabilities of selecting the paretic limb during session 2. We used two categorical independent variables: the outcome of the previous trial (success or failure), and the effector selected in the previous trial (paretic or non-paretic).

Performance in the real world pointing task is defined by the patient’s range of motion measured as the average distance covered by the paretic limb for the two targets appearing in the workspace ipsilateral to the paretic arm or the paretic workspace, for the three targets appearing at central locations (at 0°, and ±8°), and for the two targets appearing in the contralateral workspace (i.e. non-paretic workspace). In order to align workspaces across patients, we mirrored target directions for those patients with their right arm affected. We used a 2-tailed Wilcoxon signed-rank test to compare measurements obtained for each workspace and each session, during the pre and post-evaluation. We used Spearman’s rank correlation coefficient to study the dependence between outcome measurements from virtual reality and real world tasks.

To assess the patients’ responses to the questionnaire, we computed the average ratings for those statements belonging to each of the three categories, controllability, performance, and effort, attributed to each limb. Next we analyzed differences within categories, and between sessions, using a 2-tailed Wilcoxon signed-rank test. For all statistical comparisons, the significance level was set to 5 % (p = 0.05). All statistical analysis was done using MATLAB 2013a (The MathWorks, Inc. Natick, MA).

## Results

### Effects of movement amplification on reward rates

**Fig. 3:**
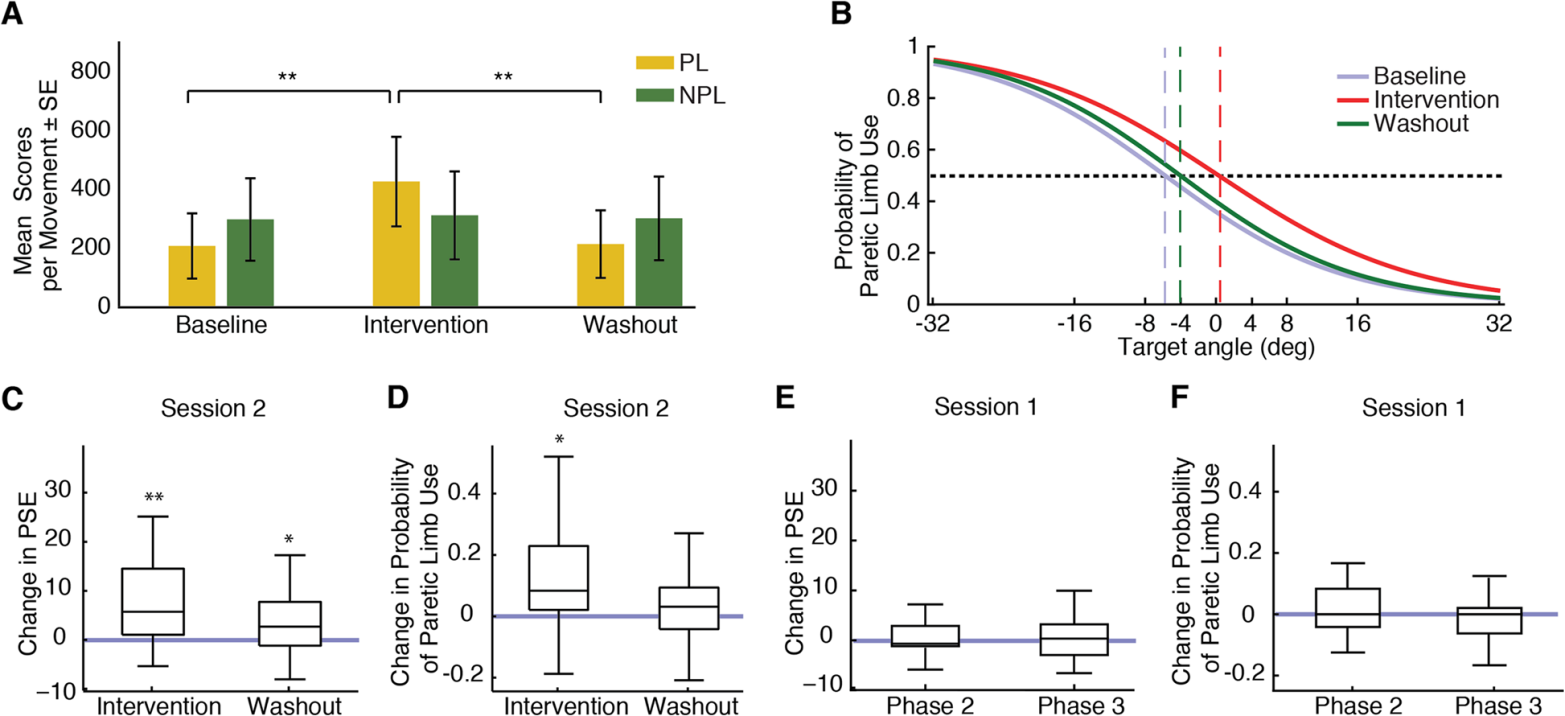
Analysis of subjects’ performance during the VR reaching task. **a**. Mean scores per phase for the paretic (yellow) or non-paretic limb (green). **b**. Logistic fit of all subject’s probabilities of paretic limb use per phase. Horizontal dashed line indicates 0.5 probabilities. Vertical dashed lines indicate target angles corresponding with PSE estimates for each phase. **c**–**d**. Change in the probability of use of the paretic limb and PSE’s respect to baseline (blue horizontal line) during session 2. **e**–**f**. Change in the probability of use of the paretic limb and PSE’s respect to phase 1 (blue horizontal line) during session 1

Our results show that the mean scores per movement of the paretic limb were significantly higher during the intervention phase when compared to baseline (p <0.001, Wilcoxon signed-rank test) (Fig. 3a). Notice that during this phase the movement of the virtual analog of the paretic limb was amplified, therefore increases in mean scores don’t necessarily reflect functional improvements. During the washout phase the performance of the paretic limb dropped 59.03 ± 23.62 % to baseline performance levels (p = 0.73, Wilcoxon signed-rank test), suggesting that changes in scores were mainly due to the amplification of virtual movements. Regarding the performance of the non-paretic limb, we observed no differences between phases (Fig. 3a). These results validate the hypothesis that a goal-oriented amplification of the trajectory of the paretic limb significantly increased the scores achieved by the subject only when using the paretic limb.

### Effects of therapy on effector selection patterns/PSE

To evaluate the effect of the virtual movement amplification in hand selection patterns, we computed the probability of selecting the paretic limb to execute the reaching movement for each phase. During the intervention phase subjects exhibited a higher probability of selecting the paretic limb when compared to baseline (p = 0.01, Wilcoxon signed-rank test). The effect disappeared during the washout phase. In order to take into account target position to analyze changes in hand selection patterns we estimated the PSE for each subject and for each phase of the task. Within-subject analysis revealed that individual PSEs were significantly shifted towards the non-paretic workspace during the intervention phase (p <0.01, Wilcoxon signed-rank test, Fig. 3d). These effects were attenuated during the washout phase but remained significantly different from baseline (p = 0.04, Wilcoxon signed-rank test, Fig. 3d). These results indicated a higher probability of selecting the paretic limb during washout phase when compared to baseline. Notice that during this phase movement amplification was no longer present. Therefore the speed, accuracy, and effort required to successfully reach a target using the paretic limb remained similar to baseline. None of these effects were observed during session 1, when amplification was never provided (Fig. 3e-f). In order to explore the stability of the effect during the washout phase, we performed a within-subjects comparison of the probability of selecting the impaired limb during the middle 24 trials and the last 24 trials within the washout. First 24 trials during washout were excluded from the analysis given that during this period visual amplifications were still partially present. We observed a slight decay in the probability of selecting the paretic limb during the washout phase, however we found no significant differences between the two sub-phases (p = 0.42, Wilcoxon signed-rank test). Hence, these results suggest that the amplification of the virtual analog of the paretic limb leads to an increase in use of a paretic limb that recalibrates the PSE.

#### Sequential analysis

**Fig. 4:**
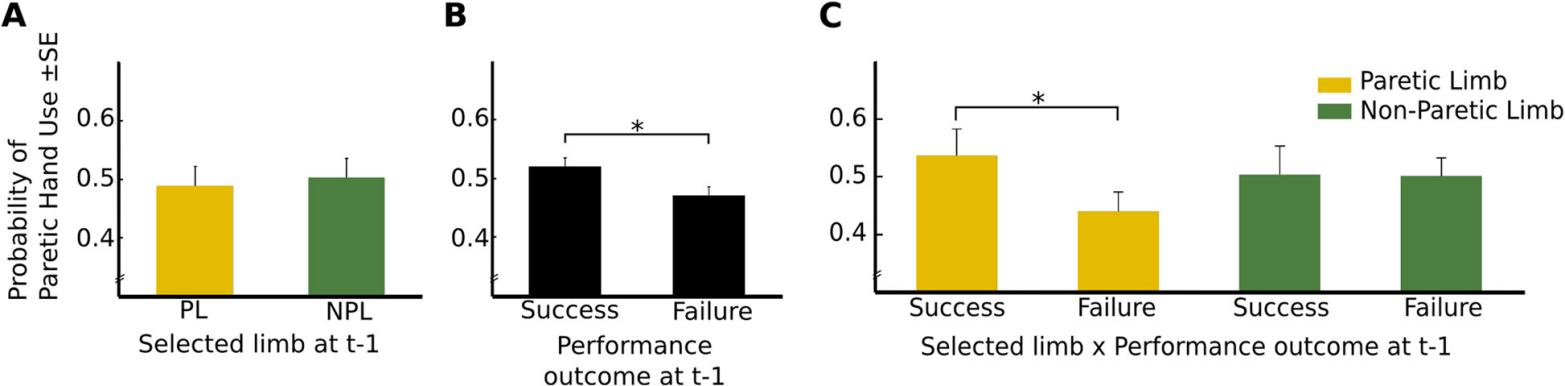
Sequential analysis of paretic hand use for session 2. **a**: Selection of either the paretic limb or non-paretic limb in the previous trial. **b**: The success or failure in the previous trial. **c**: interaction effects of both factors

In addition to target position, hand choice may be influenced by other factors, such as previous hand choice, and reinforcement history (Fig. 4). We performed a sequential analysis to quantify the probability of selecting the non-paretic hand in trial t, when in the previous trial t-1 the participant selected the paretic/non-paretic limb, or failed/succeeded to reach the target. We observed that the limb selected during the previous trial had no effect over the patient’s selection during the current trial (Fig. 4a). However, hand selection was significantly biased by performance (Fig. 4b). After a successful trial, the probability of selecting the paretic limb again was significantly higher than after an unsuccessful trial (p = 0.02, repeated-measures ANOVA). We observe that on average the increase in the probability of using the paretic limb upon success is about 3.65 % above chance level, while the decrease upon failure is -6.00 %. Next, we analyzed the interaction of both factors by quantifying the probability of selecting the paretic limb when in t-1 the patient: a) selected the paretic limb and succeeded to reach the target, b) selected the paretic limb and failed to reach the target, c) selected the non-paretic limb and succeeded to reach the target, or d) selected the non-paretic limb and failed to reach the target. Interestingly, our results show that the probability of selecting the paretic limb was strongly biased by performance (success or failure) during t-1 (p <0.05, repeated-measures ANOVA) (Fig. 4c). However, when we analyzed the probability of selecting the non-paretic limb under the same conditions, we did not find any significant effect. These results show that the condition of non-use seems to be related to the executability and desirability of the action in question rather than motoric factors per se.

#### Effects of reinforced training on real world performance

**Fig. 5:**
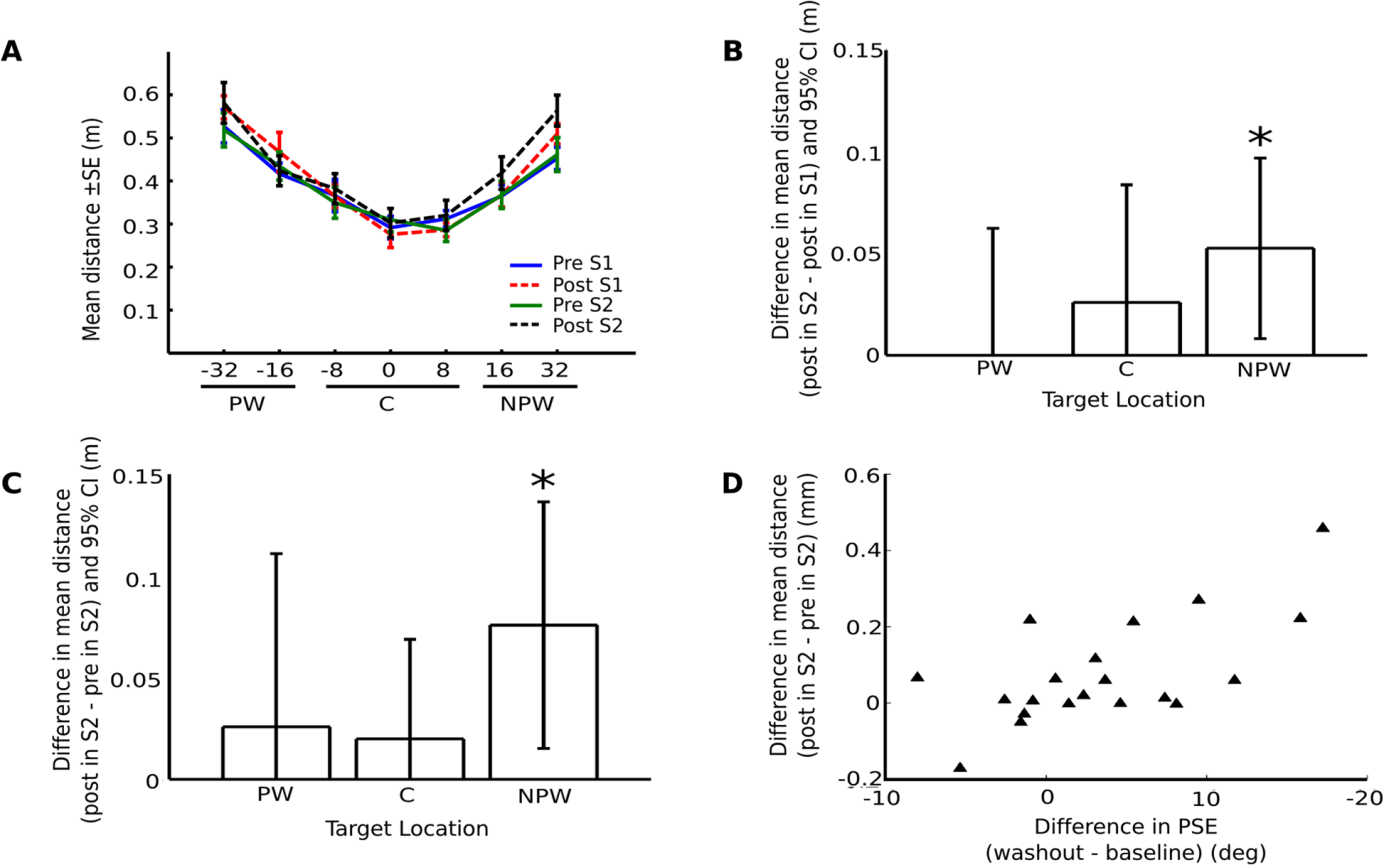
Analysis of distance covered in the real-world pointing task. **a**. Mean distance covered by the paretic limb for all trials in pre-evaluation (Pre) and post-evaluation (Post) session 1 (S1) and session 2 (S2) for each target orientation (degrees). **b**. Within-subject difference in mean distance covered between post-evaluation in sessions 1 and 2 by workspace: paretic (PW), center (C), and non-paretic (NPW). **c**. Within-subject difference between post-evaluation and pre-evaluation in session 2 by workspace. **d**. Correlation between PSE shifts during washout phase in the VR task and range of motion improvements for the paretic limb in the RW task

Within-subject analysis revealed differences in the movement amplitude of the paretic limb in the Real World Task before and after the VR task. Mean distances across target directions followed a bell-shaped profile (Fig. 5a) due to the elliptical range of motion of the arm and the starting position of the pointing task, which was placed 20 cm in front of the patient. When we segmented the data into 3 clusters depending on the target location (paretic workspace, center, or non-paretic workspace) we observed a significantly higher range of movement during post-evaluation in session 2 for the non-paretic workspace (i.e. targets at 16° and 32° from the start position) when compared to post-evaluation in session 1 (p = 0.03, Wilcoxon signed-rank test) (Fig. 5b) and pre-evaluation in session 2 (p = 0.01, Wilcoxon signed-rank test) (Fig. 5c). These gains in the range of movement correlated significantly with the individual PSE shifts observed during washout with respect to baseline (rho = 0.50, p = 0.03, Spearman’s rank correlation test) (Fig. 5d). No correlations were found for distance measurements corresponding to any of the other phases, and neither for trials in which the target was located in the center or in the paretic workspace.

**Fig. 6:**
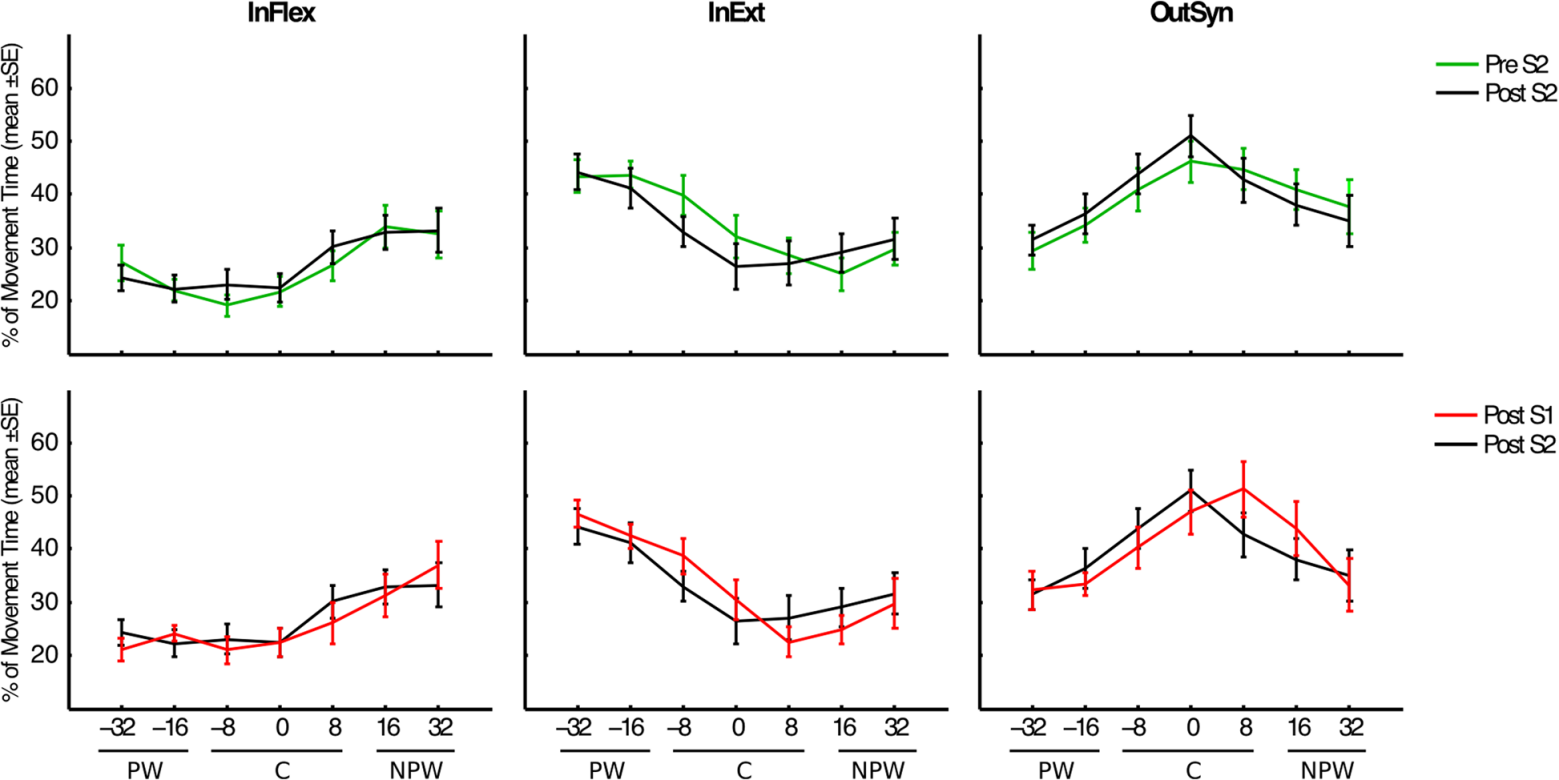
Synergistic patterns of pointing movements executed with the paretic limb. Estimation of mean percentage of movement time within the flexion synergy (InFlex), extension synergy (InExt), and out of synergistic patterns (OutSyn) for each target orientation (degrees) in the paretic workspace (PW), center (C), and non-paretic workspace (NPW)

In order to control for the introduction of compensatory movement strategies, we compared measurements of shoulder displacements across the 2 sessions and the 3 workspaces, and we observed no significant differences (p >0.71, Wilcoxon signed-rank test), indicating that observed gains in range of movement were not a consequence of wider trunk axial rotations. Besides trunk movements, differences in distance covered could also possibly be due to an exaggerated flexor synergy for the upper extremity. It is well known that after stroke some patients may exhibit an excessive excitability of ipsilateral descending pathways from the contralesional hemisphere, leading to abnormal upper limb synergies characterized by an inability to suppress antagonist muscles [22]. These abnormal flexion synergies (i.e. simultaneous shoulder abduction and elbow flexion) may be non-functional. To explore this aspect we quantified the percentage of movement within flexion synergy (InFlex), extension synergy (InExt), and out of a synergistic pattern (OutSyn) during pointing, following the method in [23]. We found no differences between evaluation phases in any of the three synergistic patterns neither for the paretic workspace nor for the non-paretic workspace (p >0.37, Wilcoxon signed-rank test) (Fig. 6). Hence, the changes we have observed are not due to a particular compensatory strategy.

#### Subjective assessment of own performance

**Fig. 7:**
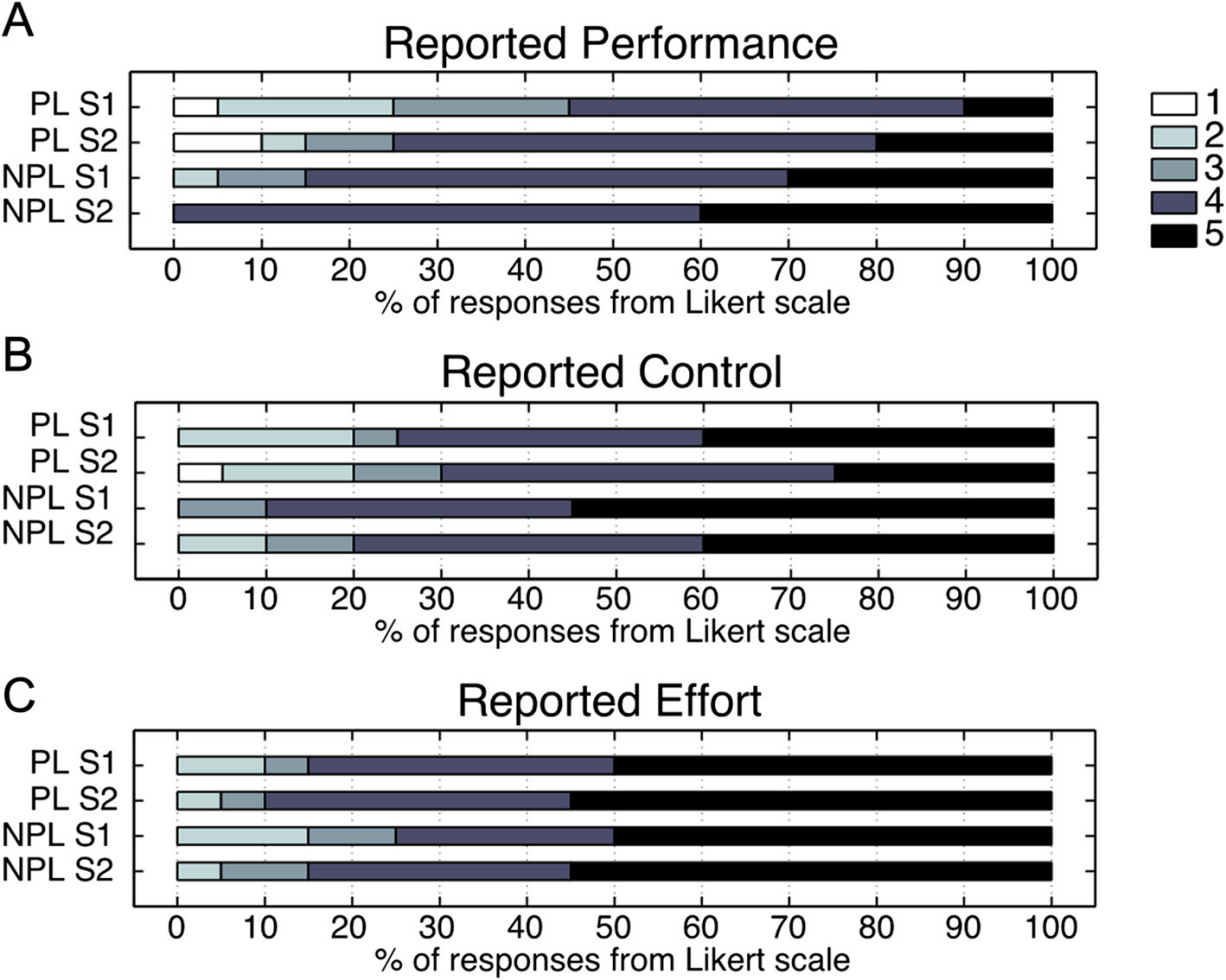
Responses to questionnaire. A divided bar chart shows median scores from a 5-point Likert scale self-report questionnaire assessing performance in virtual reality (**a**), control of the virtual limbs (**b**), and effort (**c**) for the paretic (PL) and non-paretic limb (NPL) during session 1 (S1) and session 2 (S2)

We used a questionnaire after each session to assess the awareness of the amplification of the virtual movement. Patients evaluated their own performance, effort, and the controllability for each of the virtual limbs. Patients attributed higher performance levels, lower controllability, and higher effort, to both limbs after session 2 in comparison to answers reported after session 1 (Fig. 7). However, none of these differences were significant. Patients tended to spontaneously report an internal attribution of their higher performance levels during session 2 (e.g. “It seems today I’m full of energy”, “Today I’m working hard”). Nevertheless, no patient reported awareness of the amplification of the virtual paretic limb. One of the patients commented that both virtual limbs moved faster during session 2 where only the paretic hand moved faster.

## Discussion

In this study we addressed the question of whether reinforcement-based therapies may be effective for promoting motor recovery in hemiparetic stroke patients. We amplified the movement of the paretic limb in a goal-oriented manner to facilitate performance in a reaching task. We measured changes in the probability of using the paretic limb to execute a reaching movement and assessed changes in motor performance in a real world pointing task. Results showed an increase in the probability of selecting the paretic limb for reaching towards a virtual target. After we suppressed the amplification of the virtual limb, the performance levels dropped. However patients maintained a higher probability of selecting the paretic limb when compared to baseline. Previous studies by Bonaiuto, et al. exploring the function of mirror neurons proposed that an action’s executability and desirability (i.e. expected reward) might be combined into priority for action selection [13], thus modulating hand selection patterns. Our findings suggest that the amplification of visuomotor feedback of an executed movement may reinforce the desirability of the selected action, thereby increasing its corresponding expected outcome and lowering reliance on substitutable mechanisms (e.g. using the non-paretic arm). After we suppressed the amplification of the virtual limb, the patients’ performance dropped to baseline levels, but the probability of using the paretic limb remained significantly higher. Therefore, improvements in use may not be necessarily related to increased competence, but to an increase in the patient’s expected competence (e.g. confidence) [15] and a decrease in the expected biomechanical cost (e.g. effort) of using the paretic limb [24].

Multiple studies have supported the idea that effector selection may arise from the simultaneous activation of competing action plans [25, 26]. Further supporting this theory, results from an animal experiment suggest that there is a continuous monitoring of trial history that, combined with the current perceptual evidence, modulates competition between multiple action plans [27]. Recently, Han et al. proposed a computational model of bilateral hand use in arm reaching, providing insights into a range of factors affecting recovery of arm use after stroke [28]. This model exhibits nonlinear and bistable behavior, suggesting that if performance levels reach a certain threshold,s the repeated spontaneous selection of the affected limb will engage the patient in a virtuous circle of recovery in which spontaneous arm use and motor performance reinforce each other. On this basis, CIMT may help the patient to enter this virtuous rehabilitation cycle [29]. Our observations suggest that a reinforcement-based therapy in which the patient freely chooses which hand to use to perform a specific action may have an impact on promoting the spontaneous use of the paretic limb without the drawbacks of CIMT.

Results from a sequential analysis on hand selection bias revealed that when patients succeeded with their paretic limb, the probability of selecting again the paretic limb in the next trial increased significantly. Not so for the non-paretic limb. In contrast a previous study with healthy subjects found a positive effect of reinforcement on hand selection bias when applied to either of the two hands while performing a reaching task [16]. These differences between healthy subjects and hemiparetic stroke patients may indicate that hemiparetic patients exhibit a higher sensitivity to success and failure when using their paretic limb when compared to the non-paretic limb. These results suggest that the facilitation of goal-oriented movements when freely choosing the paretic arm may be especially effective in encouraging the use of the paretic limb during post-stroke recovery.

In order to explore whether gains observed in VR transfer to real world performance, we measured motor function in a pointing task in the real word before and after performing the VR task. Our results show that after experiencing the amplification of the paretic limb in virtual reality, patients performed wider pointing movements towards targets appearing in the non-paretic workspace. When we controlled for the introduction of compensatory movements, we observed no significant differences between any of the evaluation phases. However, since the intervention phase in our experiment was short, we cannot attribute these differences in performance to actual training-dependent motor gains. Therefore, our observations suggest that repetitive exposure to goal-oriented reinforced feedback (i.e. visuomotor manipulations) may immediately increase workspace area through the introduction of new motor strategies, probably related to increased levels of motor effort. In this study we only evaluated stroke patients with restricted inclusion criteria. However the proposed principles may also be applicable to different profiles of patients, such as stroke patients with moderate cognitive impairments and severe motor impairments. Future work will address this issue and explore the long-term effects of reinforcement-based therapies based on the visual amplification of goal-oriented movements.

## Conclusions

The present study proposes that goal-oriented movement amplification in VR enhances the use of the paretic limb in hemiparetic stroke patients. However longitudinal clinical studies should validate the therapeutic effects of reinforced protocols to induce long-term functional recovery and reverse learned non-use. These new protocols may be potentially relevant for those patients with severe motor impairments, who may not be suitable for CIMT protocols. In this vein, the benefits of reinforced therapies when compared or combined with CIMT, considering the whole spectrum of dominance for each intervention, still remain unexplored.

## Additional file

Additional file 1
**Questionnaire.** Questionnaire used to assess the patient’s awareness of visuomotor manipulations and perceived performance.

## Abbreviations

VR: Virtual reality
CIMT: Constraint induced motor therapy
MNS: Mirror neuron system
DAC: Distributed adaptive control
MCA: Middle cerebral artery
RGS: Rehabilitation gaming system
PSE: Point of subjective equality
PW: Paretic workspace
C: Center of workspace
NPW: Non-paretic workspace
InFlex: Movement within the flexion synergy
InExt: Movement within the extension synergy
OutSyn: Movement out of synergy
