# Erratum to: The visual amplification of goal-oriented movements counteracts acquired non-use in hemiparetic stroke patients

**DOI:** 10.1186/s12984-015-0100-y

**Published:** 2015-11-27

**Authors:** Belén Rubio Ballester, Jens Nirme, Esther Duarte, Ampar Cuxart, Susana Rodriguez, Paul Verschure, Armin Duff

**Affiliations:** Laboratory of Synthetic Perceptive, Emotive and Cognitive Systems, Center of Autonomous Systems and Neurorobotics, Pompeu Fabra, Roc Boronat, Barcelona, Spain; Servei de Medicina Física I Rehabilitació, Hospitals del Mar I l’Esperanç, Institut Hospital del Mar d’Investigacions Médiques, Barcelona, Spain; Servei de Medicina Física i Rehabilitació Hospital Universitari Vall dHebron, Barcelona, Spain; ICREA, Institució Catalana de Recerca i Estudis Avançats, Passeig Lluís Companys, Barcelona, Spain

Unfortunately, in the original version of this article [1] the sentence "This project was supported through ERC project cDAC (FP7-IDEAS-ERC 341196), EC H2020 project socSMCs (H2020-EU.1.2.2. 641321) and MINECO project SANAR (Gobierno de España)" was missing from the acknowledgements.

The acknowledgements have been correctly included in full in this erratum.

## References

[1] Rubio Ballester B, Nirme J, Duarte E, Cuxart A, Rodriguez S, Verschure P (2015). The visual amplification of goal-oriented movements counteracts acquired non-use in hemiparetic stroke patients. J Neuroeng Rehabil..

