# Supplementary material for: The visual amplification of goal-oriented movements counteracts acquired non-use in hemiparetic stroke patients
Source: J Neuroeng Rehabil. 2015 Jun 9;12:50. doi: 10.1186/s12984-015-0039-z (PMC4460841; doi:10.1186/s12984-015-0039-z)
Supplement: Additional file 1 — Questionnaire. Questionnaire used to assess the patient’s awareness of visuomotor manipulations and perceived performance. [file 12984_2015_39_MOESM1_ESM.docx]

Questionnaire used to assess the patient’s awareness of visuomotor manipulations and perceived performance.

*1. Sometimes the right limb on the screen moved by itself.(c)*

*2. Sometimes the left limb on the screen moved by itself. (c)*

*3. Sometimes I felt the right limb on the screen was my own arm. (c)*

*4. Sometimes I felt the left limb on the screen was my own arm. (c)*

*5. It was easy to control the right limb on the screen. (c)*

*6. It was easy to control the left limb on the screen. (c)*

*7. In comparison to other patients, I think I did well this activity when using my right limb. (p)*

*8. In comparison to other patients, I think I did well this activity when using my left limb. (p)*

*9. I didn’t put much energy when using my right limb. (e)*

*10. I didn’t put much energy when using my left limb. (e)*

*11. I think I’m good at this when using my right limb. (p)*

*12. I think I’m good at this when using my left limb. (p)*

*13. I’m satisfied with how I performed with my right limb. (p)*

*14. I’m satisfied with how I performed with my left limb. (p)*

*15. I put maximum effort when using my right limb. (e)*

*16. I put maximum effort when using my left limb. (e)*

*17. It was very important for me to perform well in this activity when using the right limb. (e)*

*18. It was very important for me to perform well in this activity when using the left limb. (e)*

Each question was followed by the following set of possible answers:

*1= Totally disagree. 2=Disagree. 3=Neutral. 4=Agree. 5=Totally agree.*

*Items categories: c=control. p=performance. e=effort.*
